# Amino Acid Polymorphisms in Hepatitis C Virus Core Affect Infectious Virus Production and Major Histocompatibility Complex Class I Molecule Expression

**DOI:** 10.1038/srep13994

**Published:** 2015-09-14

**Authors:** Megumi Tasaka-Fujita, Nao Sugiyama, Wonseok Kang, Takahiro Masaski, Asako Murayama, Norie Yamada, Ryuichi Sugiyama, Senko Tsukuda, Koichi Watashi, Yasuhiro Asahina, Naoya Sakamoto, Takaji Wakita, Eui-Cheol Shin, Takanobu Kato

**Affiliations:** 1 Department of Virology II, National Institute of Infectious Diseases, Tokyo 162-8640, Japan; 2 Department of Gastroenterology and Hepatology, Tokyo Medical and Dental University, Tokyo 113-8519, Japan; 3 Center for Interprofessional Education, Tokyo Medical and Dental University, Tokyo 113-8510, Japan; 4 Laboratory of Immunology and Infectious Diseases, Graduate School of Medical Science and Engineering, KAIST, Daejeon 305-701, Korea; 5 Department of Liver Disease Control, Tokyo Medical and Dental University, Tokyo 113-8519, Japan; 6 Department of Gastroenterology and Hepatology, Hokkaido University Graduate School of Medicine, Sapporo 060-8638, Japan

## Abstract

Amino acid (aa) polymorphisms in the hepatitis C virus (HCV) genotype 1b core protein have been reported to be a potent predictor for poor response to interferon (IFN)-based therapy and a risk factor for hepatocarcinogenesis. We investigated the effects of these polymorphisms with genotype 1b/2a chimeric viruses that contained polymorphisms of Arg/Gln at aa 70 and Leu/Met at aa 91. We found that infectious virus production was reduced in cells transfected with chimeric virus RNA that had Gln at aa 70 (aa70Q) compared with RNA with Arg at aa 70 (aa70R). Using flow cytometry analysis, we confirmed that HCV core protein accumulated in aa70Q clone transfected cells, and it caused a reduction in cell-surface expression of major histocompatibility complex (MHC) class I molecules induced by IFN treatment through enhanced protein kinase R phosphorylation. We could not detect any effects due to the polymorphism at aa 91. In conclusion, the polymorphism at aa 70 was associated with efficiency of infectious virus production, and this deteriorated virus production in strains with aa70Q resulted in the intracellular accumulation of HCV proteins and attenuation of MHC class I molecule expression. These observations may explain the strain-associated resistance to IFN-based therapy and hepatocarcinogenesis of HCV.

More than 170 million people worldwide have been infected with hepatitis C virus (HCV). Of these, 60% develop chronic hepatitis, and 5–20% develop cirrhosis and hepatocellular carcinoma^1^,^2^. More than 350,000 people die from these HCV-related diseases annually. However, the mechanisms of how HCV evades host immunity and maintains chronic infection status are not fully understood. To eradicate the virus from infected patients, interferon (IFN)-based treatments have commonly been used, but they are lacking in efficacy^3^,^4^,^5^. Therefore, predictive factors for outcomes of these treatments have been investigated extensively, and several such factors have been identified, including viral load, genotypes, and polymorphisms in the virus genome. Akuta *et al.* first reported that amino acid (aa) polymorphisms (Arg/Gln at aa 70 and Leu/Met at aa 91) in the core of the genotype 1b strain were potent predictors of poor response to IFN-based therapy^6^. Subsequently, several studies revealed that the polymorphism at aa 70 is more potent than the polymorphism at aa 91 and is associated with disease progression and development of hepatocellular carcinoma^7^,^8^,^9^,^10^,^11^,^12^,^13^,^14^,^15^,^16^,^17^,^18^. Notably, this is the only polymorphism in the HCV genome that is associated with both IFN treatment failure and the establishment of hepatocellular carcinoma.

HCV is a positive-stranded RNA virus that possesses an open reading frame encoding a large polyprotein^19^. This polyprotein is cleaved by cellular and viral proteases into 10 structural and non-structural (NS) proteins^20^. Core, E1 and E2 are structural proteins and components of virus particles. The others (p7, NS2, NS3, NS4A, NS4B, NS5A and NS5B) are NS proteins that are associated with viral replication. Core forms the capsid shell to house and protect genomic RNA. It has been reported that both the interaction between core and NS5A and the localization of this structure into lipid droplets are indispensable to virus particle assembly^21^,^22^. Therefore, polymorphisms in core would affect the efficiency of various steps of the viral life cycle, particularly the viral particle assembly step. In addition to the formational role in viral particles, core has been reported to have pleiotropic effects on various host cell functions, including signal transduction, gene expression, cell cycle, hepatic steatosis and tumorigenesis^23^,^24^,^25^,^26^,^27^,^28^,^29^,^30^,^31^,^32^,^33^,^34^,^35^,^36^.

HCV evades host immune responses in several ways. In infected cells, HCV NS3 protein directly cleaves adaptor proteins that are important for IFN production^37^,^38^. In addition, HCV blocks the translation of IFN-stimulated genes by inducing the phosphorylation of protein kinase R (PKR) and eukaryotic translation initiation factor 2α^39^. Recently, we observed HCV-associated attenuation of the IFN-induced cell surface expression of major histocompatibility complex (MHC) class I molecule via PKR phosphorylation^40^.

In this study, we investigated the effects of polymorphisms in core protein (Arg/Gln at aa 70 and Leu/Met at aa 91) on the steps in the HCV life cycle and on the host immune system, particularly MHC class I molecule expression, in order to elucidate polymorphism-related resistance to IFN-based therapy, immune evasion and hepatocarcinogenesis.

## Results

### Effects of Polymorphisms in Core on Virus Phenotypes

**Figure 1:**
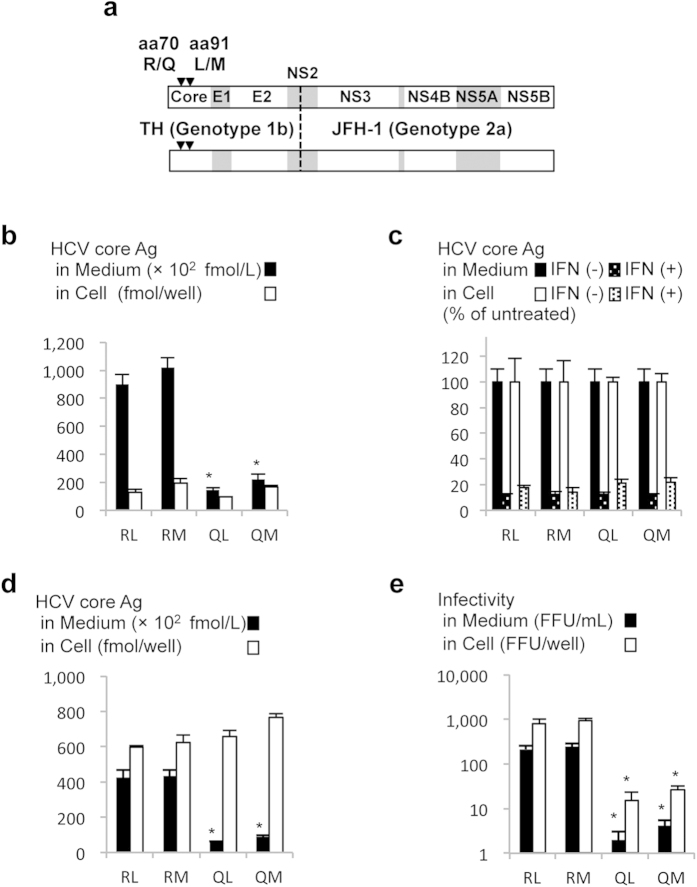
Virus production and IFN sensitivity of chimeric viruses with polymorphisms. (**a**) Schematic representation of chimeric virus (TH/JFH-1) used in this study. The virus comprises sequences of the TH strain (genotype 1b) and JFH-1 strain (genotype 2a). The junction site (indicated by dotted line) is immediately after the first trans-membrane domain of NS2. Polymorphisms R/Q at aa 70 and L/M at aa 91 are indicated by closed triangles. (**b**) Huh-7.5.1 cells were transfected with *in vitro-*transcribed RNA of TH/JFH1-RL, -RM, -QL, and -QM. HCV core Ag levels in cell lysates and medium were measured at 3 days after transfection. (**c**) To assess IFN sensitivity, transfected cells were given 10 IU/mL of IFN-α 2b at 4 h after transfection. Culture medium and cell lysates were harvested at 3 days after transfection, and the HCV core Ag levels were measured. Data are given in terms of percentage vs. untreated cells. (**d,e**) Single cycle virus production assay was performed in Huh7-25 cells transfected with *in vitro-*transcribed RNA of TH/JFH1-RL, -RM, -QL, and -QM. Culture medium and cell lysates were harvested on day 3. HCV core Ag levels (**d**) and infectivity titers (**e**) in culture medium and cell lysates were measured. Assays were performed in triplicate, and data represent means ± SD. *p < 0.05 compared with aa70R strains.

To investigate the effects of aa polymorphisms in the core of genotype 1b, we generated genotype 1b/2a chimeric HCV with polymorphisms Arg/Gln at aa 70 and Leu/Met at aa 91 (designated TH/JFH1-RL, -RM, -QL, and -QM; Fig. 1a). Three days after the *in vitro* synthesized RNA of these viral strains was electroporated into Huh-7.5.1 cells, the amounts of extra- and intra-cellular HCV core antigen (Ag) were measured in culture medium and cell lysate, respectively. Among these strains, the intracellular HCV core Ag levels were comparable (Fig. 1b). However, the extracellular core Ag levels of TH/JFH1-RL and -RM were 8.99 × 10^4^ ± 7.40 × 10^3^ and 1.02 × 10^5^ ± 7.18 × 10^3^ fmol/L, respectively, and were 4- to 7- fold higher than those of TH/JFH1-QL and -QM (1.46 × 10^4^ ± 1.32 × 10^3^ and 2.21 × 10^4^ ± 3.64 × 10^3^ fmol/L, respectively). To assess the influence of these polymorphisms on the direct anti-viral effects of IFN, we treated HCV RNA-transfected cells with 10 IU/mL (38.4 pg/mL) of IFN-α2b and measured the intra- and extra-cellular HCV core Ag levels (Fig. 1c and Supplementary Table S1). Despite clinical evidence for polymorphism-associated susceptibility to IFN treatment, the intra- and extra-cellular HCV core Ag levels of chimeric viruses with polymorphism-transfected cells were similarly reduced by IFN-α2b treatment.

### Single-cycle Virus Production Assay

For further analysis of the effects of these polymorphisms on different stages of the virus life cycle, we used a single-cycle virus production assay with Huh7-25 cells that lacked the cell surface expression of CD81. Three days after the transfection of full-length RNA of TH/JFH1-RL, -RM, -QL, and -QM, the HCV core Ag levels and infectivity titers were measured. In agreement with our observations in Huh-7.5.1 cells, the HCV core Ag levels in the culture medium were 4.8- to 6.9- fold higher in TH/JFH1-RL and -RM transfected cells than they were in TH/JFH1-QL and -QM. However, the amounts of intracellular HCV core Ag in cell lysates were similar among all strains (Fig. 1d). Both intra- and extra-cellular infectivity titers were 60- to 100- fold higher in TH/JFH1-RL and -RM than in TH/JFH1-QL and -QM, which indicated a lower efficiency for intracellular infectious virus production in strains with Gln at aa 70 (Fig. 1e, *p* < 0.05).

### Detection of Core Protein in HCV Replicating Cells

**Figure 2:**
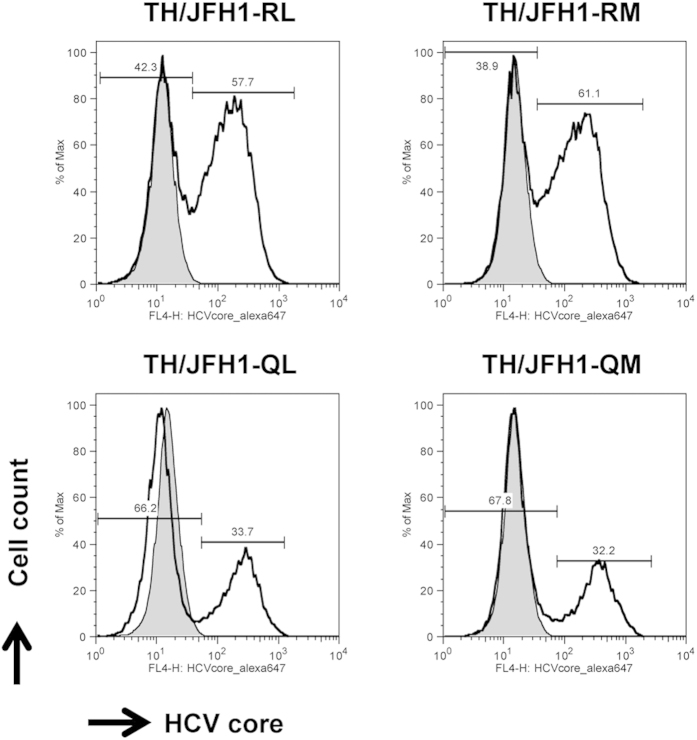
Population of HCV core-positive cells detected by flow cytometry. Huh-7.5.1 cells were transfected with RNA of TH/JFH1-RL, -RM, -QL, and -QM. Transfected cells were harvested and stained with anti-HCV core antibody. The percentages of HCV core-positive cells were counted. The shaded areas indicate mock transfections used as controls. The MFI of HCV core in the gate of core-positive cells was calculated and presented in Table 1.

In experiments with Huh-7.5.1 and Huh7-25 cells, we found that aa polymorphisms in core were associated with efficiency of intracellular infectious virus production. Thus, as a next step, we examined the effects of this polymorphism-associated inefficient virus production in host cells. After transfection of the *in vitro* transcribed RNA of TH/JFH1-RL, -RM, -QL, and -QM into Huh-7.5.1 cells, the percentages of core protein-positive cells were counted by flow cytometry, and the intracellular levels of core protein within core-positive cells were evaluated by calculating the mean fluorescence intensity (MFI). The percentages of core protein-positive cells at day 1 after transfection were similar among the strains of transfected cells, which suggested comparable levels of transfection efficiency (Supplementary Fig. S1 and Supplementary Table S2). The percentages of HCV core-positive cells were higher in cells transfected with TH/JFH1-RL and -RM than in cells transfected with TH/JFH1-QL and -QM at day 3 after transfection, thus suggesting re-infection by progeny viruses (Fig. 2 and Table 1). However, TH/JFH1-QL and -QM transfection resulted in a higher MFI of HCV core within core-positive cells than TH/JFH1-RL and -RM transfection (Fig. 2 and Table 1). In this experiment, we obtained data concerning the intracellular core levels independent from the percentage of core-positive cells by analyzing the gating of core-positive cells in flow cytometry. Taken together, these data suggest that Gln at aa 70 decreases the efficiency of intracellular infectious virus production and thus induces the accumulation of HCV core proteins in replicating cells.

### Effects of HCV Core Protein Accumulation on MHC Class I Molecule Induction

To assess the effects of accumulated HCV core protein on the host immune system and the elimination of HCV-infected cells, we evaluated the cell surface expression of MHC class I molecules by flow cytometry. Following administration of type I and II IFNs, we determined that IFN-γ most efficiently induced the cell surface expression of MHC class I molecules in Huh-7.5.1 cells (Supplementary Fig. S2). Thus, we used IFN-γ to assess the effect of core polymorphisms on MHC class I molecule expression. RNA of TH/JFH1-RL, -RM, -QL, and -QM was transfected into Huh-7.5.1 cells by using DEMRIE-C reagent. Two days after transfection, the transfected cells were treated with IFN-γ. The cell surface expression of MHC class I molecules was detected by staining with Alexa Fluor 488 conjugated anti-HLA-ABC before permeabilization. For multicolour staining, HCV core protein was detected using anti-HCV core antibody labelled with Alexa Fluor 647 after permeabilization.

**Figure 3:**
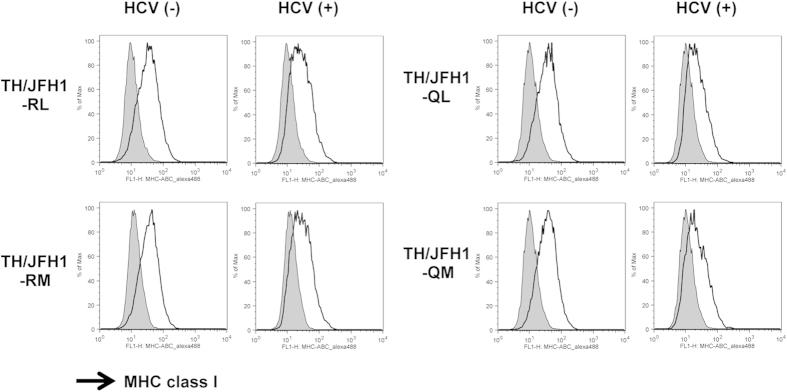
Attenuation of cell surface expression of MHC class I molecules by HCV replication. Huh-7.5.1 cells were transfected with RNA of TH/JFH1-RL, -RM, -QL, and -QM. Transfected cells were treated with 200 pg/mL of IFN-γ at day 2 and harvested at day 3 after transfection. Harvested cells were stained with anti-HLA-ABC antibody, permeabilized, and then stained with anti-HCV core antibody. The shaded areas indicate IFN-γ untreated controls. HCV core-negative and -positive populations were gated separately, and cell surface expression of MHC class I molecules was presented in HCV core-negative and -positive populations.

In HCV core-negative cells, MHC class I molecules were largely induced (Fig. 3). In contrast, in HCV core-positive cells, the induction of MHC class I molecules was suppressed. To compare the effects of aa polymorphisms in HCV core, the reduction rate of MHC class I molecule expression was calculated by using the following formula: [(MFI of MHC class I molecules in HCV-negative cells - MFI of MHC class I molecules in HCV-positive cells)/MFI of MHC class I molecules in HCV-negative cells]. The reduction rate in TH/JFH1-QL and -QM was substantially higher than that in TH/JFH1-RL and -RM (*p* < 0.05, Table 2).

### Effects of HCV Core Protein Accumulation on PKR Phosphorylation

**Figure 4:**
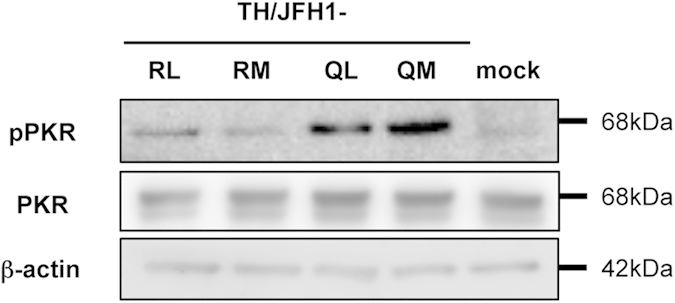
PKR phosphorylation in full-length HCV RNA transfected cells. Huh-7.5.1 cells were transfected with RNA of TH/JFH1-RL, -RM, -QL, and -QM. Transfected cells were treated with 200 pg/mL of IFN-γ 2 days after transfection and harvested at day 3. Mock transfection was used as a control. Cell lysates were subjected to immunoblotting with the indicated antibodies.

We previously reported that activation of the PKR pathway is responsible for attenuated expression of MHC class I molecules^40^. Therefore, we assessed the effects of aa polymorphisms on PKR phosphorylation. Huh-7.5.1 cells were transfected with RNA of TH/JFH1-RL, -RM, -QL, and -QM using DEMRIE-C reagent and subsequently treated with IFN-γ. Cell lysates of these treated cells were analyzed by immunoblotting. The expression levels of PKR were similar among the TH/JFH1 strains that had aa polymorphisms. However, the amounts of phosphorylated PKR were markedly higher in TH/JFH1-QL and -QM transfected cells than they were in TH/JFH1-RL- and -RM transfected cells (Fig. 4). These results indicate that the PKR phosphorylation associated with aa polymorphisms is responsible for the attenuated cell-surface expression of MHC class I molecules.

## Discussion

HCV is known to cause chronic hepatitis followed by cirrhosis and hepatocellular carcinoma. Thus, eradicating HCV is a priority for chronically infected patients. For this purpose, IFN-based therapy has played a pivotal role. However, because the efficacy of this therapy is unsatisfactory, much effort has been devoted to elucidating the mechanisms responsible for IFN resistance in this virus. The information obtained by these investigations may contribute to clarifying the strategies employed by HCV in evading the host immune system and identifying predictors of outcome in IFN-based therapy.

The aa polymorphisms in core are among the genetic factors that influence the outcome of IFN-based therapy^6^,^9^,^41^. Although the evidence accumulated from clinical studies supports a correlation between these polymorphisms and responses to IFN-based therapy, the underlying molecular mechanisms are not fully understood. This insufficient progress is partly attributable to a lack of cell-culture systems for HCV genotype1b strains. Clinical studies into these aa polymorphisms have been performed in patients infected with HCV genotype 1b strains. However, efficient cell-culture models for HCV genotype 1b strains without adaptive mutations are not currently available. JFH-1, genotype 2a, is the only strain that can efficiently replicate in cultured cells without mutations^42^, and aa polymorphisms in core are rarely observed in clinical samples of genotype 2a strains^41^.

In this study, we used a cell-culture system with JFH-1-based chimeric viruses of genotype 1b/2a to assess the function of aa polymorphisms in core of genotype 1b. By using this system, we confirmed decreased infectious virus production by chimeric viruses with aa70Q. By transfection with full-length RNA, we were able to observe higher levels of HCV core Ag in the culture medium of strains with aa70R-transfected cells compared with aa70Q. In a single-cycle virus production assay with CD81-negative cell lines, intra-cellular infectious virus production was lower in strains with aa70Q compared with aa70R. In flow cytometry, MFI of core protein staining in each core-positive cell was higher after transfection of strains with aa70Q compared with aa70R. These data suggest that the decreased virus production of strains with aa70Q is related to the intracellular accumulation of HCV proteins. Despite the apparent phenotypic association with the polymorphism at aa 70, we were unable to detect any significant differences in comparisons of the polymorphism at aa 91. Although significant differences have been found in clinical observations between patients with the polymorphism at aa 91 in several reports, other reports have been contradictory^7^,^9^,^12^,^13^,^14^,^16^,^17^.

Although we were able to detect the affected step in the HCV life cycle by studying the polymorphism at aa 70 using a system with genotype 1b/2a chimeric virus, we failed to demonstrate any differences in the direct anti-viral effects of IFN on strains with the polymorphism. The reduction rates for the extra- and intra-cellular levels of HCV core Ag were comparable between the strains with aa70R and aa70Q. These polymorphism-associated differences in susceptibility to IFN treatment have previously been reported^43^; interleukin-6 and suppressor of cytokine signalling (SOCS3)-mediated suppression of IFN signalling by strains with aa70Q or aa91M was observed in the cell culture system with JFH-1. Possible reasons for this inconsistent observation include the culture system, HCV strain (1b/2a chimeric virus vs JFH-1), and cell line (Huh-7.5.1 cells vs HuH-7 cells), although such conflicting observations have also been reported by two other groups that used genotype 1b strains^44^,^45^. In a study with the HCV genotype 1b strain infection system using human hepatocyte-transplanted mice, differences in IFN resistance were not observed among strains with aa polymorphisms in core^45^.

Instead of direct effects on susceptibility to IFN, we detected another role for aa polymorphisms in the host immune system. We previously reported that HCV replication suppresses cell-surface expression of MHC class I molecules through PKR phosphorylation^40^. Phosphorylated PKR induces phosphorylation of eIF2α to inhibit translation initiation. In the current study, we demonstrated that strains with aa70Q showed an accumulation of the HCV core protein in replicating cells because of low-efficiency virus particle production, and we showed that this accumulation enhanced PKR phosphorylation. As a result, the cell-surface expression of MHC class I molecules by IFN treatment was suppressed. HCV-specific CD8 + T cell responses are essential for the clearance of HCV infection, and MHC class I molecules play a key role in the recognition of virus-infected cells by CD8 + T cells. Reductions in MHC class I molecule expression by HCV infection may contribute to circumventing the antiviral adaptive immune responses associated with CD8 + T cells. Thus, this observation may explain one of the mechanisms for aa polymorphism-associated IFN resistance. During IFN therapy, HCV genotype 1b strains with aa70Q may remain in cells and survive by avoiding attack by CD8 + T cells. After treatment ends, surviving viruses start to replicate and propagate into other cells. HCV-positive cells evading the elimination of infected cells by T cells may be a causative factor in hepatocellular carcinoma.

In conclusion, we showed that IFN-γ-induced MHC class I molecule expression was suppressed in HCV core aa 70 variants. These findings may explain the mechanisms responsible for clinical resistance to IFN therapy and hepatocarcinogenesis.

## Methods

### *C*ell Culture

The HuH-7-derived cell lines Huh-7.5.1^46^ (provided by Francis Chisari; Scripps Research Institute, La Jolla, CA) and Huh7-25 lacking CD81 expression^47^ were used in this study. Culture conditions for these cell lines were as described previously^48^.

### Plasmid Construction and RNA Transfection

The genotype 1b/2a chimeric virus construct was generated using strains TH (Genotype 1b, accession number is AB985268) and JFH-1^49^ (Genotype 2a, accession number is AB047639) by replacing the sequences for core to the first trans-membrane domain of NS2 of JFH-1 with those of TH (Fig. 1a). The original aa residues at aa 70 and aa 91 of TH were Arg and Met, respectively. Chimeric viruses with the polymorphisms Arg/Gln at aa 70 and Leu/Met at aa 91 were generated by site-directed mutagenesis using appropriate primers (Fig. 1a).

To prepare the template for *in vitro* RNA synthesis, plasmids of these constructs were linearized at the 3′-end by XbaI digestion. HCV full-length RNA was synthesized using a MEGAscript T7 kit (Life Technologies, Carlsbad, CA) and used for RNA transfection^48^. Trypsinized Huh-7.5.1 or Huh7–25 cells were washed with Opti-MEM I reduced-serum medium (Life Technologies) and were resuspended at 3 × 10^5^ cells/mL or 1 × 10^5^/mL cells with Cytomix buffer, respectively. Ten micrograms of RNA was mixed with 400 μL of cell suspension and was transferred to an electroporation cuvette (Gene Pulser Cuvette 0.4 cm; Bio-Rad, Hercules, CA). Cells were then pulsed at 260 V and 950 μF with the Gene Pulser II (Bio-Rad). Transfected cells were immediately transferred to 6-well or 12-well plates (Corning, Corning, NY). To assess susceptibility to IFN, IFN-α2b (Intron A, MSD K.K., Tokyo, Japan) was used.

### Quantification of HCV Core Antigen

The concentration of HCV core Ag in filtered culture medium and cell lysates was measured by a chemiluminescent enzyme immunoassay (Lumipulse Ortho HCV Ag kit; Ortho Clinical Diagnostics, Tokyo, Japan) in accordance with the manufacturer’s instructions^50^,^51^.

### Titration of HCV Infectivity

Culture medium was serially diluted in 5-fold increments in complete DMEM and used to infect naïve Huh-7.5.1 cells seeded 24 h earlier in poly-D-lysine coated flat-bottom 96-well plates (Corning, Corning, NY) at a density of 1 × 10^4^ cells per well. Three days after infection, cells were stained with mouse monoclonal HCV core protein antibody (clone 2H9) for 1 h and then washed three times with PBS. Cells were then incubated with Alexa Fluor 488 anti-mouse secondary antibody (Life Technologies) for 1 h. Infectivity titer was expressed as focus-forming units per mL (ffu/mL), expressing the mean number of HCV core-positive foci detected at the highest dilutions^48^. Intracellular infectivity titer was determined as described previously^52^,^53^.

### Detection of HCV and MHC Class I Molecule Expression

To detect HCV-positive cells, we used flow cytometry (FACSCalibur; BD Biosciences, San Jose, CA, USA). The antibody used was mouse anti-HCV core 1b (Clone C7–50; Abcam, Cambridge, UK). To label this antibody, Zenon Mouse IgG Labeling Kits (Alexa Fluor 647; Life Technologies) were used. The amount of intracellular core protein was estimated independently from the percentage of core-positive cells by calculating the MFI in the gate of core-positive cells in flow cytometry.

The effects of polymorphisms on MHC class I molecule induction were then assessed. Huh-7.5.1 cells were seeded in 6-well plates at a concentration of 1 × 10^5^/mL, and 10 μg of *in vitro*-transcribed RNA was transfected with DEMRIE-C reagent (Life Technologies). To induce MHC class I molecule expression, cells were treated with 10 IU/mL (38.4 pg/mL) of IFN-α2b (Intron A), 10 IU/mL (500 pg/mL) of IFN-β (Feron, Toray Medical Co., Ltd., Tokyo, Japan), or 200 pg/mL of IFN-γ (Imunomax-γ; Shionogi & Co., Ltd., Osaka, Japan). Cell surface expression of MHC class I molecules was assessed by staining with Alexa Fluor 488 conjugated anti-HLA-ABC (clone DX17, BD Biosciences).

### Immunoblotting

Cell lysates of Huh-7.5.1 cells transfected with *in vitro*-transcribed RNA were applied to sodium dodecyl sulfate polyacrylamide gel electrophoresis and analyzed by immunoblotting. To detect PKR and its phosphorylated form, rabbit polyclonal anti-PKR (Clone E120; Santa Cruz Biotechnology, Santa Cruz, CA) and rabbit monoclonal anti-phospho-PKR, T446 (Clone E120; Epitomics, Burlingame, CA) were used. As a control for the applied proteins, β-actin was also detected with mouse monoclonal anti-β-actin antibody (Sigma-Aldrich, St. Louis, MO). To detect signals, horseradish peroxidase-conjugated anti-rabbit secondary antibody (Cell Signaling Technology, Danvers, MA) was used.

### Statistical Analysis

Assays were performed at least in triplicate. Data from repeated experiments are expressed as the means ± standard deviation (SD). Statistical analysis was performed using Student’s t-test, and *p* values less than 0.05 were considered statistically significant.

## Additional Information

**How to cite this article**: Tasaka-Fujita, M. *et al.* Amino Acid Polymorphisms in Hepatitis C Virus Core Affect Infectious Virus Production and Major Histocompatibility Complex Class I Molecule Expression. *Sci. Rep.* **5**, 13994; doi: 10.1038/srep13994 (2015).

## Supplementary Material

Supplementary Information

## Figures and Tables

**Table 1:** Percentage of HCV-positive cells and amounts of core protein.

Strains	Positive Cells (%)	MFI
TH/JFH1-RL	56.4 ± 1.2	178.1 ± 1.0
TH/JFH1-RM	60.6 ± 0.6	203.9 ± 1.6
TH/JFH1-QL	33.4 ± 0.3*	252.6 ± 1.3*
TH/JFH1-QM	31.6 ± 0.9*	396.6 ± 1.3*

MFI, mean fluorescence intensity. *p < 0.05 compared with aa70R strains.

**Table 2:** Effects of hepatitis C virus replication on MHC class I molecule expression.

	MHC Class I Molecule Expression (MFI)		Reduction Rate of MHC Class I Molecule Expression (%)
	HCV (+)	HCV (−)	
TH/JFH1-RL	34.6 ± 1.0	42.0 ± 1.6	17.7 ± 0.7
TH/JFH1-RM	38.4 ± 1.0	43.7 ± 1.0	12.1 ± 1.2
TH/JFH1-QL	29.9 ± 2.0	43.9 ± 1.4	32.0 ± 2.5*
TH/JFH1-QM	31.0 ± 0.6	44.9 ± 1.1	30.8 ± 0.5*

MFI, mean fluorescence intensity; MHC, major histocompatibility complex. *p < 0.05 compared with aa70R strains.
