# Corrigendum: Amino Acid Polymorphisms in Hepatitis C Virus Core Affect Infectious Virus Production and Major Histocompatibility Complex Class I Molecule Expression

**DOI:** 10.1038/srep17208

**Published:** 2015-12-16

**Authors:** Megumi Tasaka-Fujita, Nao Sugiyama, Wonseok Kang, Takahiro Masaki, Asako Murayama, Norie Yamada, Ryuichi Sugiyama, Senko Tsukuda, Koichi Watashi, Yasuhiro Asahina, Naoya Sakamoto, Takaji Wakita, Eui-Cheol Shin, Takanobu Kato

Scientific Reports 5: Article number: 1399410.1038/srep13994; published online: 09142015; updated: 12162015

The original version of this Article contained a typographical error in the spelling of the author Takahiro Masaki which was incorrectly given as Takahiro Masaski.

This has now been corrected in the PDF and HTML versions of the Article.

