# Supplementary material for: Amino Acid Polymorphisms in Hepatitis C Virus Core Affect Infectious Virus Production and Major Histocompatibility Complex Class I Molecule Expression
Source: Sci Rep. 2015 Sep 14;5:13994. doi: 10.1038/srep13994 (PMC4568458; doi:10.1038/srep13994)

Supplementary Information

Amino Acid Polymorphisms in Hepatitis C Virus Core Affect Infectious Virus Production and Major Histocompatibility Complex Class I Molecule Expression

Megumi Tasaka-Fujita, Nao Sugiyama, Wonseok Kang, Takahiro Masaski, Asako Murayama, Norie Yamada, Ryuichi Sugiyama, Senko Tsukuda, Koichi Watashi, Yasuhiro Asahina, Naoya Sakamoto, Takaji Wakita, Eui-Cheol Shin, Takanobu Kato.

Supplementary Figure S1. Time point analysis of HCV-positive cell population and MFI of core staining

Huh-7.5.1 cells were transfected with RNA of TH/JFH1-RL, -RM, -QL, and -QM. Transfected cells were harvested at the indicated time point and stained with anti-HCV core antibody. The percentages of HCV core-positive cells were determined. The MFI of HCV core in the gate of core-positive cells was calculated and is presented in Supplementary Table S1.

Supplementary Figure S2. Cell surface expression of MHC class I molecules induced by type I and II IFNs.

Huh-7.5.1 cells were treated with IFN-α (10 IU/mL), IFN-β (10 IU/mL) and IFN-γ (200 pg/mL) for 24 h. Harvested cells were stained with anti-HLA-ABC antibody. The shaded areas indicate IFN untreated control.

Supplementary Table S1.

IFN sensitivity of chimeric viruses with polymorphisms

Strains	HCV core Ag levels	
	IFN (-)	IFN (+)
TH/JFH1-RL		
Medium (fmol/L)	$9.0 \times 10^4 \pm 7.4 \times 10^3$	$1.1 \times 10^4 \pm 5.6 \times 10^2$
Cell (fmol/well)	127.4 ± 23.5	22.6 ± 2.0
TH/JFH1-RM		
Medium (fmol/L)	$1.0 \times 10^5 \pm 7.2 \times 10^3$	$1.3 \times 10^4 \pm 2.4 \times 10^3$
Cell (fmol/well)	195.3 ± 32.6	27.4 ± 7.1
TH/JFH1-QL		
Medium (fmol/L)	$1.5 \times 10^4 \pm 1.3 \times 10^3$	$1.8 \times 10^3 \pm 3.1 \times 10^2$
Cell (fmol/well)	94.5 ± 3.0	20.0 ± 3.0
TH/JFH1-QM		
Medium (fmol/L)	$9.0 \times 10^4 \pm 7.4 \times 10^3$	$1.1 \times 10^4 \pm 5.6 \times 10^2$
Cell (fmol/well)	164.5 ± 11.0	35.7 ± 6.1

Supplementary Table S2.

Percentage of HCV-positive cells and amounts of core protein

Strains	Positive Cells (%)	MFI
TH/JFH1-RL		
Day 1	26.9 ± 0.4	93.8 ± 0.8
Day 2	39.1 ± 0.6	147.3 ± 1.5
Day 3	52.3 ± 0.5	204.0 ± 6.1
TH/JFH1-RM		
Day 1	27.0 ± 1.2	80.8 ± 1.7
Day 2	40.3 ± 2.0	136.7 ± 0.6
Day 3	49.4 ± 0.7	215.3 ± 2.5
TH/JFH1-QL		
Day 1	25.6 ± 0.9	94.3 ± 1.1
Day 2	37.8 ± 2.4	180.3 ± 5.5*
Day 3	29.7 ± 0.9*	329.0 ± 6.1*
TH/JFH1-QM		
Day 1	26.2 ± 0.8	98.8 ± 2.1
Day 2	34.6 ± 1.9*	186.0 ± 1.0*
Day 3	29.4 ± 0.2*	300.7 ± 11.5*

MFI, mean fluorescence intensity. *$p < 0.05$ compared with aa70R strains.

Supplementary Figure S1

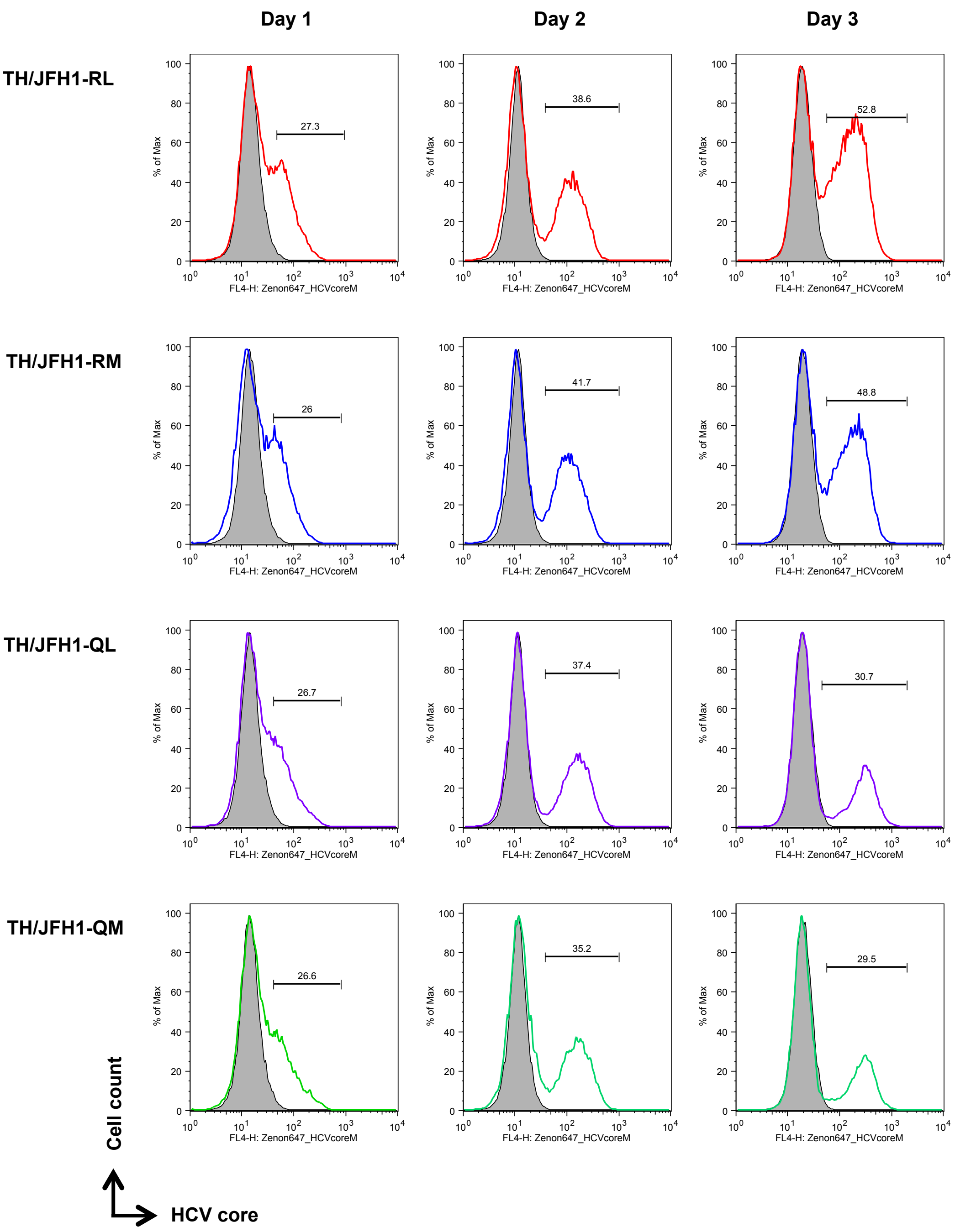

Supplementary Figure S2

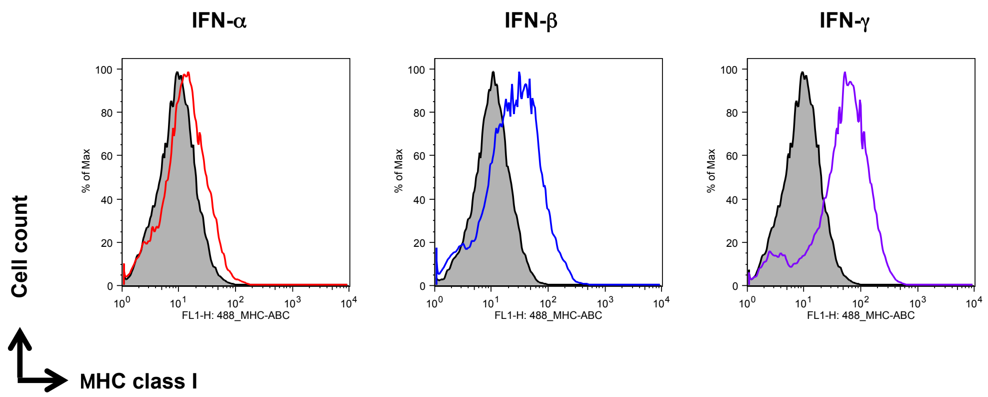

Supplement: Supplementary Information [file srep13994-s1.pdf]
